# Meeting Report from the Genomic Standards Consortium (GSC) Workshop 9

**DOI:** 10.4056/sigs.1353455

**Published:** 2010-12-04

**Authors:** Tanja Davidsen, Ramana Madupu, Peter Sterk, Dawn Field, George Garrity, Jack Gilbert, Frank Oliver Glöckner, Lynette Hirschman, Eugene Kolker, Renzo Kottmann, Nikos Kyrpides, Folker Meyer, Norman Morrison, Lynn Schriml, Tatiana Tatusova, John Wooley

**Affiliations:** 1 J. Craig Venter Institute, 9704 Medical Center Drive, Rockville, MD 20850, USA; 2 NERC Center for Ecology and Hydrology, Oxford, OX1 3SR, UK; 3 Wellcome Trust Sanger Institute, Wellcome Trust Genome Campus, Hinxton, CB10 1SA, UK; 4 Department of Microbiology and Molecular Genetics, Michigan State University, East Lansing, MI 48824, USA; 5 Plymouth Marine Laboratory (PML), Prospect Place, Plymouth PL1 3DH, UK; 6 Microbial Genomics Group, Max Planck Institute for Marine Microbiology and Jacobs University Bremen, D-28359 Bremen, Germany; 7 Information Technology Center, The MITRE Corporation, 202 Burlington Road, Bedford, MA 01730, USA; 8 Seattle Children’s Hospital, 1900 9^th^ Avenue, Seattle, WA 98101, USA; 9 DOE Joint Genome Institute, Walnut Creek, CA, USA; 10 Argonne National Laboratory, 9700 South Cass Avenue, Argonne, IL 60439, USA; 11 School of Computer Science, University of Manchester, Manchester, UK; 12 University of Maryland School of Medicine, 801 West Baltimore Street, Room 661, Baltimore, MD 21201, USA; 13 National Center for Biotechnology Information, National Library of Medicine, National Institutes of Health, 45 Center Drive, Bethesda, MD 20892-6510, USA; 14 University of California San Diego, 9500 Gilman Drive, La Jolla, CA 92093, USA

## Abstract

This report summarizes the proceedings of the 9th workshop of the Genomic Standards Consortium (GSC), held at the J. Craig Venter Institute, Rockville, MD, USA. It was the first GSC workshop to have open registration and attracted over 90 participants. This workshop featured sessions that provided overviews of the full range of ongoing GSC projects. It included sessions on  *Standards in Genomic Sciences,* the open access journal of the GSC, building standards for genome annotation, the M5 platform for next-generation collaborative computational infrastructures, building ties with the biodiversity research community and two discussion panels with government and industry participants. Progress was made on all fronts, and major outcomes included the completion of the MIENS specification for publication and the formation of the Biodiversity working group.

## Introduction

The GSC is an open-membership working body, which was formed in September 2005 [1]. The goal of this international community is to promote mechanisms that standardize the description of genomes and the exchange and integration of genomic data. Community-driven standards have the best chance of success if developed within the auspices of international working groups. Participants in the GSC include biologists, computer scientists, those building genomic databases and conducting large-scale comparative genomic analyses, and those with experience of building community-based standards. The mission of the GSC is to work with the wider community towards 1) the implementation of new genomic standards, 2) methods of capturing and exchanging metadata, and 3) harmonization of metadata collection and analysis efforts across the wider genomics community. The GSC is currently developing a range of consensus products. Formal workshops give the community a chance to meet face-to-face to exchange ideas and advance core projects. This meeting was the first to have an active recruitment for participation by previous non-participants (and thus, a formal  registration process) and was generously supported by a range of industry sponsors as well as the Gordon and Betty Moore Foundation (GBMF) and the National Science Foundation (NSF).

This workshop was recorded on video by JCVI and all talks are accessible via SciVee at URL http://www.scivee.tv/node/18902.

## Day 1: Catching up on GSC activities

The first session of the meeting provided key context for the rest of the meeting. As is tradition at past GSC meetings, the first session contained a plenary science talk and series of brief talks giving updates on key GSC activities since the last meeting. **Dawn Field (NERC Centre for Ecology and Hydrology)** chaired the session and **Karen Nelson, the Director of the J. Craig Venter Institute (JCVI)**, opened the session with a welcome to the JCVI and stressed the need for standards in genomic science and welcomed the GSC to the JCVI. She then introduced the plenary speaker of the session, her collaborator **Barbara Methe (JCVI)** who gave a high level overview of the goals of the Human Microbiome Project (HMP) [2]. The HMP project promises to generate over 1000 reference genomes in addition to 16S and metagenomic data. This large ($150 million), highly collaborative project is just one example of a growing number of megaprojects and further underscores the benefits of building consensus within large groups to bring about advancing science more efficiently.

Following the plenary talk, GSC members gave flash talks on the status of GSC core projects since last meeting. Each project has an accompanying GSC wiki page for more details. **Lynn Schriml (University of Maryland)** gave an overview of the  Research Co-ordination Network (RCN4GSC) award from the NSF to the GSC on behalf of the **PI John Wooley (University of California San Diego)**. This five-year award is being used mainly to support exchanges of young researchers between GSC labs to help implement core GSC projects. Renzo Kottmann is the first member of the GSC to undertake such an exchange, visiting both University of Maryland and Argonne National Laboratory.

**Peter Sterk (NERC Centre for Ecology and Hydrology)** and **Pelin Yilmaz (MPI-Bremen)** gave updates on the GSC’s family of minimum information checklists – MIGS/MIMS/MIENS [3,4]. Since the meeting, all have been finalized and posted to the GSC website. Work is now underway to write documentation to support full participation in usage. **Renzo Kottmann (MPI Bremen)**, the lead developer of the GSC’s Genomic Contextual Data Markup Language (GCDML) [5], confirmed that the newest checklist, MIENS, would soon be implemented and that an increasing number of groups are adopting GCDML. **Peter Dawyndt (University of Ghent)** reminded everyone of the Genomic Rosetta Stone project [6] and urged people to register their local database identifiers in the NCBI LinkOut system. **Folker Meyer (Argonne National Laboratory)** outlined the vision of the M5 platform, a roadmap for a next-generation computational ‘metainfrastructure’ that has been developed since the GSC meeting at ISMB 2009 [7]. **Norman Morrison (University of Manchester)** then reported on the further development of the Environment Ontology [8], an ontology required for compliance with the MIGS/MIMS/MIENS field ‘habitat’. **George Garrity (Michigan State University)**, the Editor-in-Chief of the GSC eJournal ”Standards in Genomic Science” (SIGS) reviewed the first nine months of SIGS since the launch of the journal in July 2009. The final speaker of the session, **Nikos Kyrpides (DOE Joint Genomes Institute)**, outlined the GSC’s proposal for a Microbial Earth Project, a coordinated effort to sequence 9,000 cultured type strains to help fill in our knowledge of the genomic make-up of the Tree of Life.

### Session II: The GSC's Standards in Genomic Sciences journal (SIGS)

This session was chaired by the Editor-in-Chief of the GSC’s journal *Standards in Genomic Sciences* (SIGS), George Garrity. The session began with an overview of the GEBA project [9] by **Hans-Peter Klenk (DSMZ)**. The GEBA project has been a major contributor of ‘short genome reports’ in SIGS and the form and content of the suite of current genome notes submissions were reviewed. This was followed by a presentation by **Dave Ussery (DTU)** on the potential for a new type of report in SIGS to cover pan-genomes, the description of many genomes from a single species. Progress on SIGS to date and prospects for its future development were then discussed by the entire workshop.

### Session III: MIGS/MIMS/MIENS compliance

Session III of the workshop was dedicated to the current status and future developments of MIGS/MIMS/MIENS compliant genome, metagenome and single sequence (marker genes) submissions. The chair Frank Oliver Glöckner kicked off this session by introducing the first three speakers. **Pelin Yilmaz (Max Planck Institute for Marine Microbiology, Bremen)** started the session with a brief history about MIENS [4] as part of the MIGS/MIMS family of checklists. The objective of MIENS is to target submissions of phylogenetic and functional marker genes from all three domains of life, from surveys to cultured organisms and independent of the sequencing platform used. The current status of the MIENS checklist version 2.1 was presented with the first MIENS compliant entries in the European Nucleotide Archive (ENA), GenBank and the Short Read Archive (SRA) available under the accession numbers GU949561.1, GU949562.1 and SRP001108, respectively. The release cycle of MIENS has been set to 12 month to facilitate tool development. Wiki pages and a ticket system will be made available for documentation, bug tracking and feature requests. The paper describing MIENS is now ready for submission. Open points for MIENS were the units for fields as well as the availability of tools for data acquisition and submission. **Guy Cochrane (European Bioinformatics Institute/European Nucleotide Archive)** underlined that the International Nucleotide Sequence Database Collaboration (INSDC) fully supports minimal standards. It is a mission of the INSDC to gather and make freely available public domain nucleotide sequencing data with comprehensive global coverage. The INSDC is involved in several minimal standards projects like MGED, CBoL, TDWG/GBIF, event-qual and ssrformat. The basic components of minimal standards from the INSDC side are Submissions, Persistence and Presentation. All components should be validated. **Ilene Mizrachi (GenBank)** emphasized that the INSDC now encourages submitters to submit MIGS/MIMS/MIENS compliant data. Compliant records are flagged with keyword, stored as structured comment in sequence records and in sample and experiment objects in SRA records. She introduced the TODS tool based on Excel sheets for the diffusion of standards and gave the first examples for MIENS compliant records in INSDC.

In the Flash session, **Renzo Kottmann (Max Planck Institute for Marine Microbiology, Bremen)** gave an update of the current status of MetaBar. Metabar is a sample inventory manager using barcodes and validated Excel spreadsheets for consistent contextual data acquisition. It is integrated into the Marine Ecological Genomics Database (MegDb) underlying the megx.net portal and has several options to export data in GCDML, structured comment, Excel and Keyhole Markup Language (KML). In the meantime MetaBar has been published [10]. **Andreas Wilke (Argonne National Laboratory)** showed the current status and planned developments to support contextual data acquisition on the MG-RAST [11] server. The MIMS checklist as well as the ontology support for Envo-Lite and a general ontology lookup tool, Terminizer [12], have been implemented. Data export in GCDML is available. Full MIENS compliance including all environmental packages and a public version of the MetaData editor will be available in the near future. **Dinos Lolios (DOE Joint Genome Institute)** showed the current status of the Genomes Online Database (GOLD) [13]). A phylogenetic distribution as well as the journal where the genome has been published can now be visualized. Forty-five genomes published in SIGS are already available. Additional contextual data can be added to the project using the GOLD web interface. **Linda Amaral-Zettler (Woods Hole Laboratory)**, the program manager for the International Census of Marine Microbes (ICoMM, U.S.A.) (http://icomm.mbl.edu/microbis) and PI of the Microbial Inventory Research Across Diverse Aquatic Long Term Ecological Research Sites (MIRADA-LTERS) (http://amarallab.mbl.edu) projects at the Marine Biological Laboratory of Woods Hole gave an overview of the progress on metadata and contextual data capture for both of these projects. ICoMM and MIRADA-LTERS are currently moving towards MIENS compliance with as many of their 454 pyrosequencing projects as possible. Examples can be seen by visiting the following URL: http://icomm.mbl.edu/microbis/project_pages/details/index.php?project=PML. **Jim Cole (Michigan State University**) from the Ribosomal Database Project [14], reiterated the commitment of the RDP to the GSC standards. Specifically, he indicated that the *my*RDP SRA PrepKit will be upgraded to the new MIENS standard as soon as it is finalized. **Owen White (University of Maryland)** from the Human Microbiome Project Data Analysis and Coordination Center [2] explained that their reference genomes samples sequenced in the program are marked up with metadata compliant with the MIENS standard. On the clinical side metadata is collected for patient samples and the plan is to map these data to corresponding MIENS fields. **Chris Hunter (European Bioinformatic Institute)** gave a flash update on the proposal from EBI for the generation of a metagenomic data-collection and analysis portal. The portal will aid submission of GSC-compliant metadata together with raw sequence data to the SRA, as well as providing researchers with tools to analyze their datasets and search collections of metagenomic data experiments. Finally, the EBI aims to enable/encourage data exchange with other metagenomic service providers such as CAMERA and MG-RAST. **Susanna Sansone (European Bioinformatics Institute)** gave an update on the Investigation/Study/Assay (ISA) infrastructure [15]. This is the first general-purpose and *freely available desktop software suite* targeted to curators and experimentalists that (1) assists in the reporting and local management of *experimental metadata* from studies employing one or a combination of technologies, (2) empowers users to take up community-defined minimum information checklists and ontologies, including MIGS/MIENS and EnvO and (3) formats studies for submission to a growing number of international public repositories like ENA/SRA (genomics), PRIDE (proteomics), and ArrayExpress (transcriptomics). The ISA software suite comprises several platform-independent Java-based components for local use, including a relational database. ISA tools can work independently, or as unified system. All components have GUIs, and command lines. **Inigo San Gil (Long Term Ecological Research Network Office / National Biological Information Infrastructure)** presented steps taken towards the harmonization of the existing ecological metadata standards and the specifications developed by the Genomics Standards Consortium, specifically GCDML and the MIENS checklist. The LTER Network Office has approved merging both XML schemas, the Ecological Metadata Language (EML) and GCDML, and to start testing the functionality of these information transport vehicles. Inigo also announced the use case of Linda Amaral-Zettler’s MIRADA project to test these goals and tools based on the Drupal content management system created for the actual metadata editor user interface.

### Plenary Talk by David Lipman

At this point in the agenda, the GSC included the first plenary talk from an external speaker. David Lipman, Director of the NCBI, spoke on the subject of *Communicating Scientific Knowledge: Is progress lagging?*

Over the last 30 years, advances in DNA sequencing technology and other high throughput methods have led to exponential growth in biomedical data. Right from the beginning, the leadership at the National Institutes of Health and other major funding agencies promoted explicit policies on data sharing and also supported centralized databases for molecular data. The biomedical research community has become an intensive user of these resources and directly contributes their own data - often before publication. This virtuous circle has led to an increased rate of biological discovery. Fortuitously, these advances have taken place during the rise of the internet, further promoting researchers' ability to access and analyze scientific data. Indeed, the Web has dramatically enhanced the ability of all sectors of society to communicate and to share information. Despite the progress in data sharing and analysis, and despite the increased capabilities of the Web, our ability to communicate biomedical knowledge has not kept pace. By communicating knowledge I mean publishing biomedical research papers, reference materials and educational content. I will discuss these issues and suggest some possible solutions.

In terms of solutions, he discussed the new project *PloS Currents* and his efforts in launching the influenza section of this publication. *PloS Currents Influenza* is a moderated collection for rapid and open sharing of useful new scientific data, analyses, and ideas. Future *PloS Currents* sections will provide a new forum for rapid publication of research data changing the face of scholarly publishing of analyses of topical data.

### Session IV: Linkages between the GSC and the biodiversity community

Recognizing that genomic standards have an increasingly important role to play in biodiversity, this “biodiversity” session was designed to start building capacity between the GSC and key partners in the wider biodiversity research community. GSC members already working in the area of biodiversity research included Linda Amaral-Zettler and Inigo San Gil. Invited external speakers came from the Global Biodiversity Information Facility (GBIF), The National Ecological Observatory Network (NEON), the Global Genome Initiative, GGI. The chair, **Norman Morrison (NERC Environmental Bioinformatic Centre and The University of Manchester)**, charged the speakers with a set of questions. Each speaker had 10 minutes to give an overview of key activities within each of these large collaborative projects, in particular highlighting the standards work being done and emphasizing any molecular work. Speakers were also asked to suggest how each project might work with/contribute to/work within the GSC - in particular towards uptake of MIGS/MIMS/MIENS or contributions to any implementation activities. **Inigo San Gil (LTER Network Office / National Biological Information Infrastructure)** kicked off the session with an update on the activities of the Long Term Ecological Research Network. The LTER Network has been collecting an extensive and diverse array of valuable environmental data stretching back for over 20 years. Inigo highlighted the need for interoperability between the EML and GCDML and the requirement for better tools for standards compliant metadata capture. Significantly, Inigo pointed out that the DataONE initiative has been funded by the NSF to provide universal access to data about life on earth and the environment that sustains it. **Linda Amaral-Zettler (Marine Biology Laboratories)** presented the latest developments of the Microbial Inventory Research Across Diverse Aquatic LTERs (MIRADA) Project. One of the major goals of the project is to provide the data, infrastructure and analysis tools to interpret microbial biodiversity in an environmental context. One of the key challenges in a project as complex as this is to make appropriate use of environmental meta-data. Linda highlighted that MIRADA has been making use of both the MIENS standard and EnvO ontology in their data collections and they have been surfacing their data using the Microbial Oceanic Biogeographic Information System (MICROBIS). **Giri Palanisamy (Oak Ridge National Laboratory)** was unable to attend, but Inigo San Gil agreed to present his talk on The Global Biodiversity Information Facility (GBIF). The main message of Giri’s slides was for the establishment of strong links between the genomics and biodiversity communities going forward in order to maximize the interoperability of specimen level and genomics data, promoting the use of persistent identifiers and shared common vocabularies. **Rachel Gallery (National Ecological Observatory Network (NEON))** presented the goals and work program of the recently established National Ecological Observatory Network (NEON). The mission of NEON is to enable understanding and forecasting of climate change, land use change and invasive species on continental-scale ecology by providing infrastructure (both information and physical) to support research in these areas. Rachel highlighted that NEON was in the process of establishing its cyber infrastructure and would recommend GSC standards to the team. **Sean Brady (National Museum of Natural History, Smithsonian Institution)** introduced the goals of the Global Genome Initiative (GGI). The GGI is a highly ambitious initiative to preserve high quality genomic grade tissues that broadly span species biodiversity in order to provide knowledge for future generations, dubbed “Life on Ice”. **Shaochuan Li (Beijing Genomics Institute)** presented some of the astonishing volumes of high-throughput sequencing capacity at the BGI and highlighted some of the cutting edge microbiome research work the institute has been involved in. Shaochuan also highlighted that the BGI was always very keen to hear from interested partners with important scientific problems. **Katja Schulz (Smithsonian Institution)** gave a brief overview of the application of biodiversity data standards in the Encyclopedia of Life (EoL) project. Katja demonstrated that EOL has been linking to external content providers of molecular information, in particular the species pages have links to barcode information for species identification.

In conclusion, the session was a positive, important first step in building concrete links to the biodiversity community. Importantly, the GSC Biodiversity working group was established at this meeting, and now has a significant number of members (GSC Biodiversity Working Group Page). One speaker commented at the end of the session that the GSC seemed to be a very organized and effective  group that was progressing forward while listening to the community and that they would be keen to attend further meetings.

## Day 2

### Session V: Unifying concepts in genomic annotation standards

This session, chaired and organized by **Nikos Kyrpides (DOE Joint Genomes Institute)**, carried on the themes from similar sessions at GSC6 and GSC 8. Increasingly, the GSC is beginning to shift from a focus of capturing top-level metadata used to describe genomes and metagenomes to thinking about how we can better describe genomic annotations. **Nikos Kyrpides** started with a review of potential ways of standardizing gene calling. **Tatiana Tatusova (NBCI, Refseq)** gave an overview of the NCBI annotation workshop that had been held back-to-back with GSC 9. In the annotation workshop, groups worked on a variety of issues including minimum information about genome annotations, Standard Operating Procedures (SOPs), and standardization of annotations specifically for viral genomes. **Owen White (University of Maryland)** then gave an overview of his vision for a future ”Critical Assessment of Functional Annotation Experiment (CAFAE) competition. A first time attendee of a GSC meeting, **Peter Karp (SRI)** then gave an exploratory talk entitled *Should metabolic pathway inference be standardized?*

### Session VI: Metagenomes, Metadata, Meta-analysis, Models and MetaInfrastructure (M5) Roadmap

**Folker Meyer (Argonne National Laboratory)** chaired a session dedicated to the working group he chairs within the GSC, that of M5. The session was intended to invite new people into the M5 working group and stimulate discussion about future developments of data sharing standards particularly in the area of metagenomics.

The field of metagenomics sustains dramatic growth, fueled by our ever-growing ability to obtain data. The data analysis bottleneck represents a major a limitation for the community. Sharing data, metadata and expensive computational results derived from those data will become a requirement for biology in the future. Folker outlined plans for “outsourcing” data set analysis to large computational centers, in particular, the maintenance (updating) of existing large datasets. In a second presentation, **Jeff Grethe (University of California San Diego)** representing CAMERA [16] outlined the existing workflow systems that will allow a system-independent description of computations performed on the data that will allow users to trust computed results. Both presenters outlined the current state of development and options for further development. In a third talk **Guy Cochrane (European Bioinformatics Institute)** summarized the plans for metagenomic analysis at the EBI.

### Session VII: Industry and Government Involvement in the GSC

This session was represented the first coordinated attempt by the GSC to reach out to industry and government to help shape its future vision.

#### Industry Panel

The Industry Panel was chaired by **Jack Gilbert (Plymouth Marine Laboratory; Argonne National Laboratory).** Industries, especially those involved in marketing sequencing platforms and informatics, have a considerable role to play in helping to proactively promote data standards. The GSC envisages this role to be through the directed delivery of workflows and SOPs relating to sequence production and analysis. Importantly, vendors could take a lead in providing a road map for the collection of metadata associated with ‘omic-projects. This panel provided a forum for representatives from industrial sponsors of GSC9 to give their take on GSC activities and the role of industry in the development and promotion of data standards. The panel was open to community questions and interaction following the presentations.

**Asim Siddiqui (Life Technologies)** provided a thorough overview of the new SOLiD platforms which are set to provide a significant increase in data production. He discussed the BAM format for standardization of sequence alignment formats, suggesting that it had been adopted as the central file format for the Bioscope 1.2 analysis pipeline. He highlighted the need for open governance of platforms for standardization of data. He iterated that the General Feature Format  (GFF) provided the best standard for defining genomic variants when comparing strains. **David Dailey (Illumina)** re-iterated the need for standardization of data formats. As the dominant sequencing platform, Illumina highlighted the need to be able to compare sequencing output across platforms so as to enable better and more sophisticated alignments for genome resequencing projects. **Jim Knight (454)** highlighted the basic sequencing formats of FASTA, Quality Scoring and ACE, and detailed their development of SFF file format submission to the Short Read Archive (SRA). He also suggested that the sequence alignment/map file formats  SAM and BAM would be readily supported to achieve more sophisticated data integration across the pyrosequencing platforms and alternatives. **Brian Bramlett (LuxBio)** gave a compelling talk about the need for standardization of the SOP for bioinformatic data pipelines and outlined the ongoing initiatives at Lux Bio to achieve this goal. **Saul Kravitz (CLC-Bio)** outlined similar ideas and went on to discuss the need for establishing provenance in all pipelines. **Justin Johnson (EdgeBio)** highlighted the role of individual industrial groups in providing customers with access to all sequencing platforms. He suggested that democratization of sequencing only went so far. Instead, being able to access various sequencing platforms in one place, and multi-task these technologies to provide a holistic analysis of a defined problem was paramount to the customer. He coined the term Next Generation Sequencing as a Service (NGSaaS). He went on to highlight the role of bioinformatics initiatives such as BioLinux and M5 in the cloud computing revolution, especially regarding the need for data sharing and utilization protocols and infrastructure.

Following the individual talks, the panel fielded questions from the floor. Most of these questions pertained to determining the role of industry in defining the solutions for the upcoming data-storm. Asim Siddiqui outlined the role of industry in helping to manage the data from production to analysis and beyond to sharing and archiving. The panel was very receptive to questions from the floor and demonstrated a genuine interest in adopting the ideals and policies of the GSC, as well as helping to define them. This was especially true for file transfer formats.

#### Government Panel

The government panel was chaired by **Lynette Hirschman (MITRE)** and **Dan Drell (DOE).** Multiple government agencies have a stake in the development of widely-adopted standards, capture of multi-omics and ecological data and metadata, and sharing across community boundaries. The government panel provided a forum for representatives from various government agencies to present and discuss their perspectives on critical issues for the advancement of science at the intersection of genomics, metagenomics, ecology, bioenergy, biosecurity, and environmental science.

The panel included seven participants from six US governmental organizations: **Matt Davenport (DHS S&T)**, **Maria Giovanni (NIAID)**, **Gopal Gopinathrao (FDA)**, **Matt Kane (NSF/DEB)**, **Victor Pollara (Noblis supporting DTRA)**, **Marc Salit (NIST) and Sylvia Spengler (NSF/CISE).** Each panelist spoke for a few minutes about directions of their respective organizations and priorities in the area of data and meta-data standards in support of cross-disciplinary data sharing. Overall, the panelists were enthusiastic about the mission and activities of GSC. Several panelists emphasized the importance of standards in enabling community data sharing activities. The panelists pointed out that different agencies have different missions, e.g., DOE is now heavily focused on microorganisms related to bioenergy, while NIAID is focused on disease-causing agents, DHS S&T is focused on biothreats, and NSF funds basic research in biology and at the intersection of biology and computer science. While the staff at these organizations talk to each other, and participate in cross-agency working groups (such as the Interagency Pathogen Sequencing Working Group, chaired by Dan Drell), there is currently no good mechanism to provide coordinated funding for basic infrastructure serving multiple missions, such as MIENS (Minimal Information about ENvironmental Sequences), which can serve the metagenomics, biodiversity and microbiome communities.

After brief presentations by the panelists, there was a general discussion among the panelists and the audience. The feedback from the panel was that 1) the GSC is doing an outstanding job and needs to continue its excellent work; 2) the GSC should continue to increase its visibility through publications and community discussions; and 3) to obtain funding from US agencies, the GSC will need to write proposals specifically tailored to the mission of specific agencies.

## Day 3

### Session VIII: How to get involved in the GSC

This session was designed to give all the GSC working group leaders a chance to highlight key areas of activity for the short- and long-term and attempt to recruit new members. The session was chaired by Renzo Kottmann, lead developer of the GCDML project. Dawn Field kicked off the session by outlining how to get in involved in the GSC. For each project people were encouraged to:

- adopt GSC standards, such as the metadata standards MIGS/MIMS/MIENS [suggested by Peter Sterk (NERC CEH) and Pelin Yilmaz (MPI-Bremen)]
- extend and adopt GCDML for markup and exchange uses and give feedback [suggested by Renzo Kottmann (MPI-Bremen)]
- submit their identifiers to NCBI Linkout so that they can be picked up by the  Genomic Rosetta Stone for resolution of the different identifiers used by different databases [suggested by Peter Dawyndt (University of Ghent)]
- participate in the M5 project by joining regular teleconferences, help develop and implement MTF pipelines and submit Standard Operating Procedures to SIGS [suggested by Folker Meyer (Argonne National Laboratory)]
- use and contribute to the Environmental Ontology [suggested by Norman Morrison (University of Manchester)]

The session ended with a one slide presentation of the Open Microbiome Initiative (OMI). As the GSC is an open membership organization, it was emphasized that everyone who is willing to contribute can join by contacting a member.

### Session IX: Development of GSC Strategy

During this session the GSC Board had a closed meeting and participants were encouraged to network to discuss specific projects. There were two formal break-out groups that carried on more detailed discussions. The first was the MIENS group, led by Pelin Yilmaz (MPI-Bremen). This group finalized the MIENS specification. In particular, there was discussion about whether to require specific units for reporting of measurements and observations in MIENS environmental packages. In the end, units are left out for the time being with the recommendation that all units should be written following the International System of Units (SI) conventions. A voting session resolved a few minor outstanding issues and led to new fields being added and/or changed accordingly. The MIENS manuscript was discussed in detail. The second group included speakers from the biodiversity session, organized by Norman Morrison (University of Manchester). From this break out group came the decision to establish a formal biodiversity working group within the GSC.

## Conclusions

This was the largest GSC meeting to date, attracting a large number of new attendees. It was also the first to directly engage representatives from industry and from government to help shape the future of the GSC. Key outputs included the completion of the MIENS checklist and formation of the biodiversity working group.
